# Hormone-controlled cooperative binding of transcription factors drives synergistic induction of fasting-regulated genes

**DOI:** 10.1093/nar/gkac358

**Published:** 2022-05-12

**Authors:** Dana Goldberg, Meital Charni-Natan, Nufar Buchshtab, Meirav Bar-Shimon, Ido Goldstein

**Affiliations:** Institute of Biochemistry, Food Science and Nutrition. The Robert H. Smith Faculty of Agriculture, Food and Environment. The Hebrew University of Jerusalem. POB 12, Rehovot 7610001, Israel; Institute of Biochemistry, Food Science and Nutrition. The Robert H. Smith Faculty of Agriculture, Food and Environment. The Hebrew University of Jerusalem. POB 12, Rehovot 7610001, Israel; Institute of Biochemistry, Food Science and Nutrition. The Robert H. Smith Faculty of Agriculture, Food and Environment. The Hebrew University of Jerusalem. POB 12, Rehovot 7610001, Israel; Institute of Biochemistry, Food Science and Nutrition. The Robert H. Smith Faculty of Agriculture, Food and Environment. The Hebrew University of Jerusalem. POB 12, Rehovot 7610001, Israel; Institute of Biochemistry, Food Science and Nutrition. The Robert H. Smith Faculty of Agriculture, Food and Environment. The Hebrew University of Jerusalem. POB 12, Rehovot 7610001, Israel

## Abstract

During fasting, hepatocytes produce glucose in response to hormonal signals. Glucagon and glucocorticoids are principal fasting hormones that cooperate in regulating glucose production via gluconeogenesis. However, how these hormone signals are integrated and interpreted to a biological output is unknown. Here, we use genome-wide profiling of gene expression, enhancer dynamics and transcription factor (TF) binding in primary mouse hepatocytes to uncover the mode of cooperation between glucagon and glucocorticoids. We found that compared to a single treatment with each hormone, a dual treatment directs hepatocytes to a pro-gluconeogenic gene program by synergistically inducing gluconeogenic genes. The cooperative mechanism driving synergistic gene expression is based on ‘assisted loading’ whereby a glucagon-activated TF (cAMP responsive element binding protein; CREB) leads to enhancer activation which facilitates binding of the glucocorticoid receptor (GR) upon glucocorticoid stimulation. Glucagon does not only activate single enhancers but also activates enhancer clusters, thereby assisting the loading of GR also across enhancer units within the cluster. In summary, we show that cells integrate extracellular signals by an enhancer-specific mechanism: one hormone-activated TF activates enhancers, thereby assisting the loading of a TF stimulated by a second hormone, leading to synergistic gene induction and a tailored transcriptional response to fasting.

## Graphical Abstract

**Graphical Abstract:**
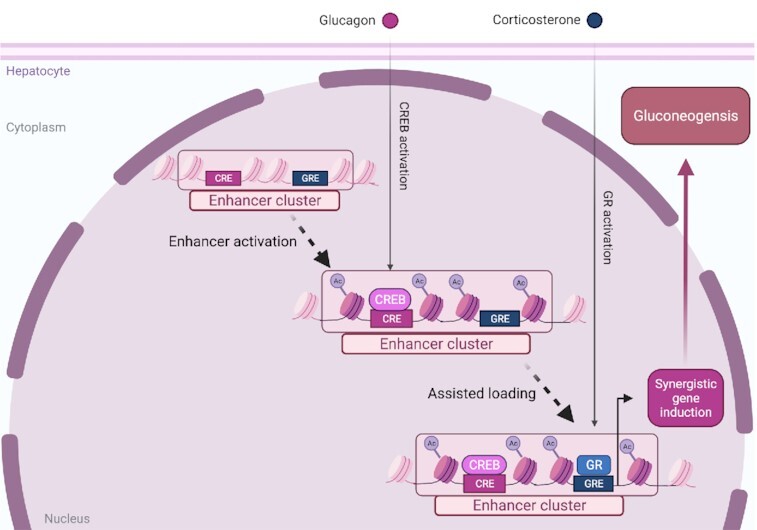
During fasting, glucagon and glucocorticoids regulate gene expression. We found that the glucagon-CREB axis activates enhancers, thereby assisting GR loading onto these enhancers, leading to synergistic induction of gluconeogenesis genes.

## INTRODUCTION

During fasting, significant hormonal and metabolic changes occur to ensure sufficient energy supply to cells. Insulin levels decrease while glucagon and glucocorticoids levels increase (1–8). Lipolysis in adipose tissue leads to release of free fatty acids reaching the liver (9) and protein breakdown in muscle generates amino acids that are also taken up by the liver (10). In turn, the liver is responsible for producing fuel in the form of glucose and ketone bodies to supply extrahepatic tissues. Hepatocytes produce glucose by glycogen breakdown and by gluconeogenesis - the de novo synthesis of glucose from non-carbohydrate precursors (mainly amino acids coming from muscle). Due to hepatic glucose production, circulating glucose levels are only mildly reduced during fasting (4,8,11). In addition, hepatocytes oxidize free fatty acids to acetyl CoA, serving as a precursor for ketone body production (12). Free fatty acids also play a role in augmenting the glucose production capacity of the liver (13).

Gluconeogenesis is potently stimulated by the two fasting hormones glucagon and glucocorticoids. The effects of these hormones on hepatocytes are widespread and are in part mediated via transcriptional regulation (14). Glucagon, a peptide hormone secreted from alpha cells in pancreatic islets, binds the glucagon receptor on hepatocytes and stimulates signal transduction cascades mediated by cAMP and calcium. These cascades elicit various biological effects in hepatocytes including enzyme activity regulation, metabolite uptake and transcriptional regulation (15,16). The major transcription factor (TF) activated by glucagon is cAMP responsive element binding protein - CREB (17). Upon glucagon stimulation, CREB regulates gluconeogenic genes (18) as well as genes responsible for providing gluconeogenic precursors (19). In addition to glucagon, glucocorticoids are also secreted during fasting and dramatically affect hepatic gene expression and metabolism. Glucocorticoids bind the glucocorticoid receptor (GR), a TF that regulates various hepatic genes, including gluconeogenic genes (20–22).

While other TFs were shown to play a role in regulating gluconeogenic gene expression (14,23), CREB and GR were among a group of four TFs that were found to play a genome-wide role in the transcriptional response to fasting and were shown to preferentially bind hundreds of hepatic enhancers promoting the fasting response (3). Among these four TFs, only CREB and GR are potently stimulated by fasting hormones. Therefore, CREB and GR are central players in the fasting response due to their dynamic activation following fasting, their widespread binding within fasting enhancers and their effect on gene expression during fasting.

Early studies have shown that co-infusion of glucagon and glucocorticoids leads to above-additive glucose production (24,25), suggesting cooperation between these two hormones. Indeed, studies have shown that the two hormones cooperate in a synergistic manner to induce several genes related to gluconeogenesis (26–29). We have previously shown that GR promotes the binding of CREB at some enhancers via an ‘assisted loading’ mechanism, a model of TF cooperativity in which a TF binds to an enhancer and activates it, thereby making the region more amenable to binding of a second TF (30). We found that following glucocorticoid stimulation, GR binds enhancers, activates them and facilitates the subsequent binding of CREB to the same enhancers. This is mediated by increase in chromatin accessibility and enhancer activity markers (3). Assisted loading was described by us and others in several scenarios to lead to synergistic gene induction (3,31,32). Indeed, enhancer-centered TF cooperativity is emerging as a principal mode of gene regulation [for examples, see (33–39)]

As detailed above, the cooperation between glucagon and glucocorticoids is well known. However, there are many open questions which we set out to answer in this study: What is the genome-wide transcriptional effect of glucagon and glucocorticoids on hepatocytes? Do the two hormones cooperate to regulate only a handful of genes or is it a cooperation module broadly affecting the transcriptional response to fasting? Is the crosstalk between the two hormones only cooperative or also antagonistic? What are the determinants of CREB-GR cooperation in terms of enhancer environment, enhancer selectivity, motif strength and TF binding?

## MATERIALS AND METHODS

### Reagents

Glucagon 100 nM (Ray biotech, cat# 228-10549-1), corticosterone 1 μM (Sigma-Aldrich, cat# 50-22-6).

### Biological resources

Primary hepatocytes were isolated from male, 8–10 weeks-old mice (strain C57BL/6JOlaHsd)

### Primary mouse hepatocytes

Isolation and plating of primary mouse hepatocytes (PMH) was performed as detailed in our published protocol with no modifications (40). Three hours after plating, media was changed to Williams E media (ThermoFisher Scientific, cat# 12551032). All hormone treatments were performed 18 h after plating in Williams E media except for adenovirus infection experiments, detailed below. Male, 8–10 weeks-old mice (C57BL/6JOlaHsd) were used for isolations. All animal procedures are compatible with the standards for care and use of laboratory animals. The research has been approved by the Hebrew University of Jerusalem Institutional Animal Care and Use Committee (IACUC). The Hebrew University of Jerusalem is accredited by the NIH and by AAALAC to perform experiments on laboratory animals (NIH approval number: OPRR-A01-5011).

### RNA preparation, reverse transcription and quantitative PCR (qPCR)

Total RNA was isolated from primary mouse hepatocytes using NucleoSpin kit (Macherey-Nagel cat# 740955.25) according to the manufacturer's protocol. For qPCR, 1 μg of total RNA was reverse transcribed to generate cDNA (Quantabio cat# 76047–074). qPCR was performed using a C1000 Touch thermal cycler CFX96 instrument (Bio-Rad) using SYBR Green (Quantabio cat# 101414–276). Gene values were normalized with a house keeping gene (*Rpl13*). The primers indicated ‘nascent’ were designed to amplify nascent transcripts (i.e. the amplified region span exon-intron junctions) as a proxy for transcription and in order to avoid confounding post-transcriptional events. The sequences of primers used in this study are:

*Rpl13* - Fwd: AGCCTACCAGAAAGTTTGCTTAC, Rev: GCTTCTTCTTCCGATAGTGCATC

*Ahr (nascent) -* Fwd: AGGATCGGGGTACCAGTTCA, Rev: ATGTGCCGTATATCAGGCGG

*Fh1 (nascent) –* Fwd: AGGTGTCGAACTCTACACGGA, Rev: GCTGGTCAGAGTTTGTTTGCTTT

*Fosl2 (nascent) –* Fwd: CGTCGAATCCGGAGGGAGA, Rev: GCATAATGTCGACCCATGTCC

*Gpcpd1 (nascent) –* Fwd: CCAGCGCTTCTTCCACTCTC, Rev: GACCCACCTTTGACAAGTCCT

*Ppp1r3g (nascent) –* Fwd: GGATGCACTTCGCTCGATTG, Rev: ATAGCTTTGATCCACCCCGC

*Mt1 (nascent) –* Fwd: CTGCTCCACCGGTAAGACTC, Rev: CAAGCCTCTACAACTCGGGG

*Nr3c1 –* Fwd: CTCCCCCTGGTAGAGACGAA, Rev: TTGACTGTAGCTCCACCCCT

*Creb1 –* Fwd: TGTAGTTTGACGCGGTGTGT, Rev: TCCACTCTGCTGGTTGTCTG

### Chromatin immunoprecipitation (ChIP)

PMH were treated with hormone combinations for 1 h. Then, cells were cross-linked with 1% formaldehyde (Electron Microscopy Sciences, cat# 15714) for 10 min at room temperature and quenched with 0.125M glycine. Crosslinked samples were washed in phosphate buffered saline (PBS), resuspended in ChIP lysis buffer (0.5% SDS, 10mM EDTA, 50mM Tris-HCl pH8) and sonicated (Bioruptor Plus, Diagenode) to release 100–1000 bp fragments. Samples were diluted 1:5 with ChIP dilution buffer (170mM NaCl, 17mM Tris-HCl pH8, 1.2mM EDTA, 1.1% Triton x-100, 0.01% SDS). Antibodies (4 μl per 2 mL sample) against H3K27ac (Active Motif, cat# 39133), or GR (Cell Signaling Technologies, cat# 3660) were conjugated to magnetic beads (Sera-Mag, Merck, cat# GE17152104010150) for 2 h at 4°C. Chromatin was immunoprecipitated with antibody-bead conjugates for 16 h at 4°C. Immunocomplexes were washed sequentially with the following buffers: low-salt buffer (0.01% SDS, 1% Triton x-100, 2 mM EDTA, 20mM Tris-HCl pH8, 150mM NaCl), high salt buffer (0.01% SDS, 1% Triton x-100, 2mM EDTA, 20mM Tris-HCl pH8, 500mM NaCl), low salt buffer and TE buffer (10m MTris-HCl, 1mM EDTA pH8). Chromatin was de-proteinized with proteinase K (Hy Labs, cat# EPR9016) for 2 h at 55°C and de-crosslinked for 12 h at 65°C. DNA was subsequently phenol-chloroform purified and ethanol precipitated.

The sequences of primers used in ChIP-PCR are:

Unassisted site – Fwd: TCACCCTGTGCCAGGACCAA, Rev: TGGGGAAGGGTGAGCAAGCT

Assisted site 1 – Fwd: ATTGCCTGCTGGCGACTAAA, Rev: GGATCCAAGTCCAAGGCACA

Assisted site 2 – Fwd: GTGCCGACAAACCTCTACTTG, Rev: AAGAGTGCTACCTGTGACGAC

### RNA-seq and ChIP-seq

For quality control of RNA yield and library synthesis products, the RNA ScreenTape and D1000 ScreenTape kits (both from Agilent Technologies), Qubit RNA HS Assay kit, and Qubit DNA HS Assay kit (both from Invitrogen) were used for each specific step. mRNA libraries were prepared from 1 μg RNA using the KAPA Stranded mRNA-Seq Kit, with mRNA Capture Beads (KAPA biosystems, cat# KK8421). ChIP DNA libraries were prepared using the KAPA HyperPrep Kit (KAPA biosystems, cat# KR0961). The multiplex sample pool (1.6 pM including PhiX 1%) was loaded on NextSeq 500/550 High Output v2 kit (75 cycles) cartridge, and loaded onto the NextSeq 500 System (Illumina, San Diego, CA, USA), with 75 cycles and single-read sequencing conditions.

### Protein preparation of whole cell lysates and nuclei/cytoplasmatic fractionation

To separate nuclear and cytoplasmic fractions, we followed the REAP protocol (41) with modifications - while the cells were still adhered to the plate, plasma membrane was disrupted using 0.1% NP-40 (Tergitol, Sigma-Aldrich, cat # NP40S) diluted in PBS and protease inhibitor cocktail, (Sigma-Aldrich cat# P2714). Cells were scraped, collected to a tube and incubated on ice for 10 min. Samples were centrifuged (4°C, 2000rpm, 3 min.) and supernatant (the cytoplasmic fraction) were transferred to a new tube. The pellet (nuclei fraction) was lysed with RIPA buffer (50mM tris-Cl, 150mM NaCl, 1% triton, 0.5% sodium deoxycholate, 0.1% SDS) followed by sonication (twice for 30 sec, ‘High’ setting, BioRuptor Plus, Diagenode) and centrifugation (4°C, 2000rpm, 10 min.). Supernatant is the nuclei fraction. For whole cell lysates, RIPA was added directly on adherent cells followed by scraping and centrifugation (4°C, 2000rpm, 10 min.), supernatant is the whole cell lysate.

### Western blot

Protein samples were loaded on 12% polyacrylamide SDS gels. Proteins were transferred (Trans Blot Turbo, Bio-Rad; cat# 1704158) to a nitrocellulose membrane (Trans-Blot Turbo Transfer Pack, Bio-Rad; cat# 1704158), blocked for 1 hour with 5% low-fat milk, and incubated for 16 h with primary antibody (GAPDH Santa Cruz Biotechnology cat# 365062; GR Cell Signaling Technologies cat# 3660; histone H3 Cell Signaling Technologies cat# 14269; Vinculin Cell Signaling Technologies cat# 13901; CREB Cell Signaling Technologies, cat# 9197) diluted 1:1000 in solution (tris-buffered saline 0.5% Tween, 5% bovine serum albumin). Membranes were incubated with secondary peroxidase AffiniPure goat anti-rabbit immunoglobulin G (1:2000, Jackson Laboratory, cat# 111–035-144) or anti-mouse (1:10000, Jackson Laboratory; cat# 115–035-146) for 1 h, followed by washes and a 1-minute incubation with western blotting detection reagent (Cytiva Amersham ECL prime, cat# RPN2232). Imaging and quantification were done with ChemiDoc (Bio-Rad).

### Adenovirus infection

Three hours after plating, PMH were infected with adenovirus (25 × 10^6^ PFU/mL). After 24 h, hormones were added for downstream experiments. Ad-DN-CREB was kindly provided by Charles Vinson (National Cancer Institute, USA).

### Sequencing data analyses

Fastq files were mapped to the mm10 mouse genome assembly using Bowtie2 (42) with default parameters. Tag directories were made using the makeTagDirectory option in HOMER (43). H3K27ac GR and CREB peaks were called using MACS2 (narrowPeak option) (44). All site overlaps were performed by the MergePeaks option in HOMER. Distance between sites was measured by the annotatePeaks option in HOMER (-p option). Selected gene loci were visualized by the integrated genome browser (IGV) (45).

### Differential gene expression

Differential gene expression was evaluated by DEseq2 (46) via the HOMER platform under default parameters. Genes were determined as differentially expressed between two conditions if they pass these cutoffs: fold change ≥ 1.5, adjusted p value ≤ 0.05. As part of our definition of synergistic induction, we calculated the sum of increased gene expression of the two single treatments by adding the RPKM values of the two single treatments. We compared the sum of single treatments to the RPKM value of the dual treatment (for full details of synergistic definition, see Results section).

### *k*-means clustering

All genes induced in at least one treatment (fold change ≥ 1.5, adj. p value ≤ 0.05) were included in the analysis. The normalized tag counts of each gene were used for the analysis. Morpheus (https://software.broadinstitute.org/morpheus) was used to cluster genes (*k* = 5).

### H3K27ac ChIP-seq analyses

Peak-calling was performed by MACS2, sites common to at least two replicates were merged and ENCODE blacklisted sites were omitted. Differential enhancer activity (DEseq2 fold change ≥ 1.5, adj. p value ≤ 0.05) was measured in all conditions as compared to the non-treated control. De novo motif enrichment analysis was performed using the findMotifsGenome option in HOMER (parameter -size given). The entire enhancer landscape (all H3K27ac sites across all conditions) was used as background to account for possible sequence bias. When the analysis was repeated with automatically-generated background (which matches GC content) the rank of GRE and CRE did not change. All motifs with p value ≤ 1^–10^ are shown.

### GR ChIP-seq analyses

Peak-calling was performed by MACS2, sites from both replicates were merged and ENCODE blacklisted sites were omitted. Differential binding was determined by DEseq2 via the HOMER package (default parameters, except norm2total option which was applied as is needed in TF ChIP-seq). Assisted and unassisted definitions - assisted sites were defined as GR binding sites (GRBS) showing an increase (DEseq2 fold change ≥ 1.5, adj. p value ≤ 0.05) in dual-treated cells compared to corticosterone-treated cells. Unassisted sites were defined as corticosterone-increased GRBS that are not further increased in the dual treatment. De novo motif enrichment analysis was performed using the findMotifsGenome option in HOMER (parameter -size given). All motifs with p value ≤ 1^–10^ are shown.

### Aggregate plots and box plots

Tag distribution around peak center or transcription start site (aggregate plots) were analyzed using the annotatePeaks option in HOMER (parameters: -size 8000 -hist 10). Tag density (box plots) was analyzed using the annotatePeaks option in HOMER. In GR ChIP and Dnase-seq, tag density of +/- 200 bp around the center was analyzed (parameter: -size 400 -noann). In H3K27ac ChIP, tag density of +/- 500 bp around the center was analyzed to account for the spread signal of H3K27ac (parameter: -size 1000 -noann). In all cases the plotted data is an average of all replicates.

### Enhancer cluster analyses

Enhancer clusters were analyzed in the vicinity of 91 assisted sites and 91 randomly-selected unassisted sites. Dnase hypersensitive sites that are found in fasted mice (3,19) and are located 12.5kb upstream and downstream of assisted or unassisted GRBS were isolated. GRBS with at least two Dnase hypersensitive sites in the +/- 12.5kb region were further analyzed. Motif searches and H3K27ac quantification within enhancer clusters included only hypersensitive regions and not intermediate inaccessible regions between enhancer units.

### Motif occurrences

The occurrence of GREs and CREs was found using the annottatePeaks option in HOMER (parameter: -size given). HOMER motifs used: cre.motif, gre.motif. Log odds ratio was reduced by 3 to allow near-consensus motif detection (https://doi.org/10.1101/2021.04.10.438998).

### Analyses of published data

In addition to data generated in this study, we used data that was previously published by us (3) and others (47). We analyzed CREB ChIP-seq from PMH treated with glucagon for 1 h as well as three different genome-wide outputs (RNA-seq, DNase-seq and GR ChIP-seq) from mice. Male C57Bl/6 mice (8–10 weeks old) were either fasted for 24 h or fed ad libitum. Food was removed at the beginning of the inactive phase, when lights went on in the animal facility (i.e. zeitgeber time 0, ZT0). This was done to prevent the reported disruption of circadian clock when mice encounter fasting in the beginning of the active phase (48). Livers from both the fasted and fed groups were collected at the same time (ZT0). All data is available in Gene Expression Omnibus (GEO) under accession number GSE72087. Lists of fasting-induced genes and fasting-activated Dnase hypersensitive sites were previously generated and are openly available (19). These lists were generated as follows: differential gene expression or Dnase accessibility were evaluated by DEseq2 (46) via the HOMER platform under default parameters. Genes or Dnase sites were determined as differentially expressed between two conditions if they pass these cutoffs: fold change ≥ 1.5, adjusted p value ≤ 0.05. CCCTC-binding factor (CTCF) ChIP-seq sites in mouse livers were downloaded from GSE104129 (47). CTCF binding in livers from ad libitum fed mice (collected at ZT22 and ZT10) was analyzed, combined and visualized on IGV.

### Statistical analyses

All conditions in all of the described experiments were performed in three biological replicates except for GR ChIP-seq which was performed in two biological replicates. Error bars represent standard deviation of biological replicates. In pairwise comparisons, statistical significance was determined by a two-tailed, unpaired t test. In multiple comparisons, statistical significance was determined by ordinary one-way ANOVA (comparisons were made between all conditions, only statistically significant comparisons are depicted in most cases). One asterisk denotes statistical significance of p value ≤ 0.05. Two asterisks denote statistical significance of p value ≤ 0.01. Three asterisks denote statistical significance of p value ≤ 0.001. Four asterisks denote statistical significance of p value ≤ 0.001. ‘ns’ denotes statistical significance of p value ≥ 0.05.

## RESULTS

### The crosstalk between fasting hormones is gene-specific, with both antagonistic and synergistic interactions

**Figure 1.**
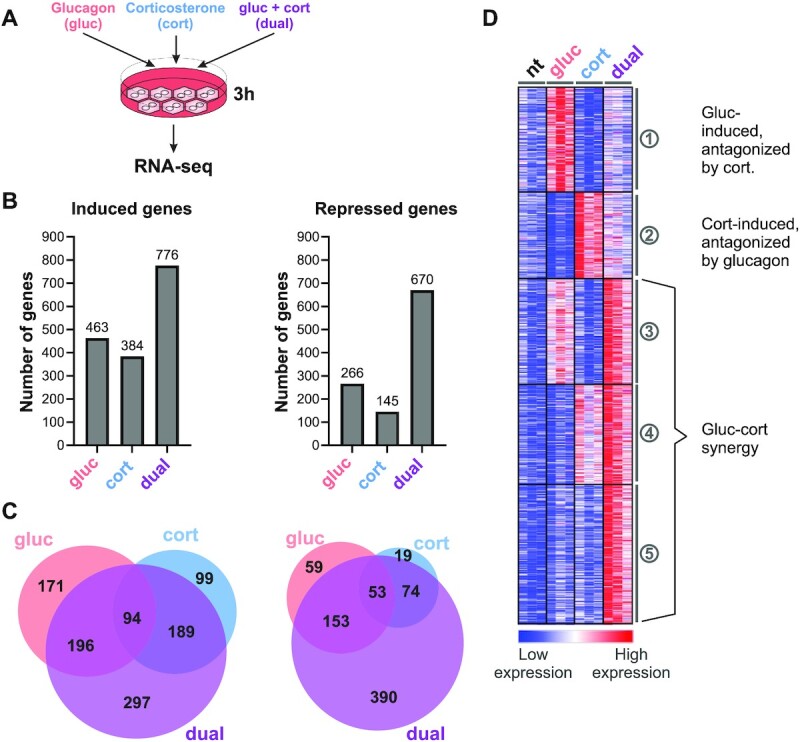
Transcriptomic profiling reveals intricate crosstalk between glucagon and corticosterone. (**A**). Scheme of experimental setup. Primary mouse hepatocytes were treated with either glucagon (100 nM), corticosterone (1 μM) or both in a dual treatment for 3 h. Then, RNA was extracted and sequenced via RNA-seq. (**B**).The number of induced and repressed genes following each treatment as compared to the non-treated control (fold change ≥ 1.5, adj. *P* value ≤ 0.05). (**C**). The overlap in the identity of induced and repressed genes between the different treatments is shown, revealing substantial overlap in the transcriptional programs of glucagon and corticosterone. Also, a considerable number of genes is only regulated in the dual treatment. (**D**)*. k*-means clustering of induced genes reveals complex crosstalk between glucagon and corticosterone, with both antagonistic and synergistic effects on gene expression (*k* = 5). (nt – non-treated; gluc – glucagon; cort – corticosterone).

We aimed to find what is the nature and extent of glucagon and glucocorticoids crosstalk in regulating hepatic gene expression. We treated primary mouse hepatocytes (PMH) with glucagon and corticosterone (the major glucocorticoid found in mice), either alone or in a dual treatment for 3 h and analyzed their transcriptome using RNA-seq (Figure 1A). The 3 h time point was selected in order to capture mostly primary transcriptional effects rather than secondary ones. After determining differential gene expression as compared to the non-treated control, we found overt gene regulation by a single treatment of either glucagon or corticosterone. This was evident in the numbers of induced and repressed genes (Figure 1B, Supplementary Table S1). These findings show that glucagon and corticosterone have a prominent, rapid and widespread effect on hepatic gene expression. When comparing the two sets of genes regulated by each single hormone treatment, we found a significant overlap. This shows that although the two hormones initiate utterly different signaling pathways and activate different TFs, the transcriptional programs imposed by them resembles each other considerably (Figure 1C).

While the pairwise comparisons above are useful in assessing the extent of gene regulation, it is less suitable for uncovering potential crosstalk between the two hormones. To discern the dominant crosstalk patterns between glucagon and corticosterone, we performed *k*-means clustering on all genes induced in at least one treatment (Figure 1D). The resulting clusters revealed two major crosstalk modes between glucagon and corticosterone. Clusters 1 and 2 show antagonism between the two hormones whereby one hormone led to gene induction that was dampened by the second hormone in the dual treatment. Conversely, in clusters 3, 4 and 5 the two hormones augmented each other's effect, leading to stronger gene induction in the dual treatment compared to the single treatments. This pattern was most prominent in cluster 5 where an apparent synergistic effect is seen in which the dual treatment leads to markedly higher induction compared to either glucagon or corticosterone treatments alone. This is in line with the finding that many genes were only induced in the dual treatment and not in the single treatments (Figure 1C).

**Figure 2.**
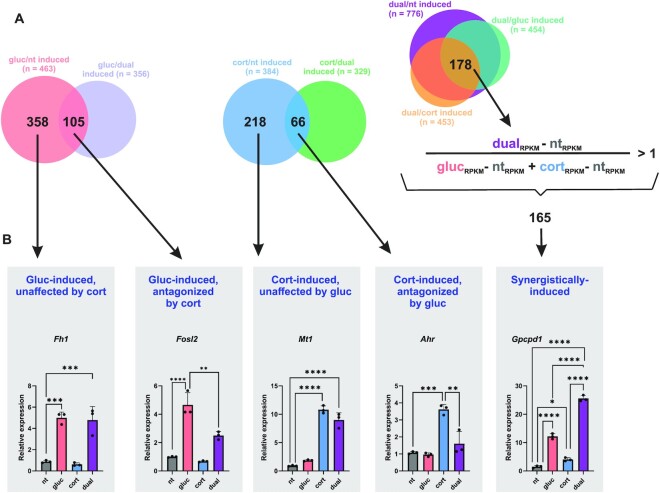
Reciprocal antagonism between fasting hormones coincides with synergistic gene induction. (**A**). The relationship between glucagon and corticosterone was defined with clear cutoffs: (i) A glucagon-induced gene antagonized by corticosterone must meet 2 criteria: induction by glucagon compared to the non-treated control and induction by glucagon compared to the dual treatment. (ii) A corticosterone-induced gene antagonized by glucagon must meet 2 criteria: induction by corticosterone compared to the non-treated control and induction by corticosterone compared to the dual treatment. (iii) A synergistically-induced gene must meet 4 criteria: induction by the dual treatment compared to the non-treated control, compared to glucagon and compared to corticosterone. Moreover, the gene expression increase in the dual treatment must be higher than the sum of increased gene expression of the two single treatments. (**B**). An example of a gene from each group is shown. Gene expression was determined by qPCR. (nt – non-treated; gluc – glucagon; cort – corticosterone; RPKM – reads per kilobase per million).

While antagonistic and synergistic expression patterns are evident from the *k*-means clustering analysis, we wanted to more robustly define antagonism and synergism using fixed parameters and statistical tests. Antagonism between glucagon and corticosterone was defined as a gene induced by the single treatment compared to both the non-treated control and the dual treatment. For example, a glucagon-induced gene whose induction is dampened in the dual treatment will be determined as antagonistic. Based on these criteria, we found 105 glucagon-induced genes that are antagonized by corticosterone. Reciprocally, 66 corticosterone-induced genes are antagonized by glucagon (Figure 2A, Supplementary Table S1). To find what are the cellular pathways that antagonistic genes participate in, we performed pathway enrichment analysis using GeneAnalytics (49). We found that the glucagon-induced genes which corticosterone antagonizes participate in pathways involving transforming growth factor β signaling and immune-related pathways rather than metabolic pathways. In contrast, corticosterone-induced genes antagonized by glucagon are related to lipid and bile metabolism (Supplementary Table S2).

The *k*-means clustering pointed to a prominent synergistic effect of the two hormones. Synergy is defined as an effect of two conditions that is greater than the sum of both conditions tested individually. To clearly determine synergy in our dataset, we set several complementing criteria, all of which must be met for a gene to be determined as synergistic: the gene is induced in the dual treatment compared to the: **(a)** non-treated control, **(b)** the glucagon single treatment and **(c)** the corticosterone single treatment. **(d)** In addition, for a gene to be defined as synergistic, its increase in expression in the dual treatment must be higher than the *sum* of increases in the two single treatments combined. Thus, only genes that pass three pairwise comparisons (both statistically and with a fold change cutoff) and show a fold change in the dual treatment that is higher than the sum of the two single treatments were defined as synergistic. Strikingly, even under these strict criteria, 165 genes were found to be synergistically induced by glucagon and corticosterone, showing that synergy is a central crosstalk mode for these two hormones (Figure 2A, Supplementary Table S1). In summary, these results show that the crosstalk between glucagon and corticosterone leads to a complex gene induction pattern that could be divided to 3 main patterns: (i) Genes induced by one hormone with no effect by the second hormone. (ii) Genes induced by one hormone with this induction dampened by the second hormone in a dual treatment. (iii) Genes synergistically induced by both hormones whereby the induction in the dual treatment is greater than the sum of the effect of both single treatments. A representative gene from each group is shown in Figure 2B (profiled via quantitative PCR, qPCR).

To explore which group of genes resembles the transcriptional program at play in the liver during fasting in vivo, we compared all gene groups to genes induced in mouse liver following 24 h of fasting [previously published by us, (3)]. We found that only 17–29% of antagonistic genes are also induced in fasted mice and 32–43% of genes induced in either treatment (single or dual treatment) are fasting-induced in vivo. Synergistic genes had the highest overlap with in vivo fasting-induced genes with 56% (n = 92) of synergistic genes induced in the liver during fasting (Supplementary Table S1). These results suggest that the majority of antagonistic genes are not part of the hepatic fasting response while the synergistic group of genes more closely resembles the gene grogram induced during fasting.

To get insights into the biological processes regulated by synergistically-induced genes, we performed pathway enrichment analysis. The synergistic group of genes was highly enriched in pathways related to gluconeogenesis and catabolic routes leading to gluconeogenesis (i. e. amino acid metabolism and urea cycle; Supplementary Table S2). This finding is in accordance with our previous studies showing that a dual treatment of glucagon and corticosterone leads to a synergistic increase in gluconeogenesis from both pyruvate and amino acid precursors (3,19). This also aligns with the observed synergistic glucose production following infusion of both hormones to dogs and humans (24,25). Thus, the cooperative gluconeogenic effect of glucagon and corticosterone is associated with an extensive synergistic gene induction program of gluconeogenic genes.

### The transcriptional response to fasting hormones is accompanied by robust dynamics in enhancer activity

**Figure 3.**
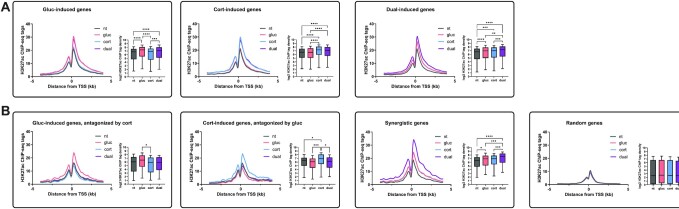
Dynamics in promoter-proximal H3K27ac accompanies gene induction patterns. (**A**). H3K27ac signal was measured in regions surrounding transcription start sites (TSS) of hormone-induced genes. Hormone treatment leads to enhancer activation around hormone-induced genes in a pattern that is concordant with gene induction. (**B**). H3K27ac signal was measured in regions surrounding TSS of antagonistic and synergistic genes. Enhancers adjacent to antagonistic genes show increased activity in the single treatment while in the dual treatment their activity is reduced back to basal levels. In contrast, enhancers adjacent to synergistic genes show increased activity in the single treatments, with further augmentation in the dual treatment. Thus, enhancer activation and gene induction are completely concordant, suggesting gene induction is driven by enhancer activation. (nt – non-treated; gluc – glucagon; cort – corticosterone).

The above findings suggest that the pro-gluconeogenic transcriptional response occurring during fasting is dominated by glucagon-corticosterone cooperation. We have previously shown that the transcriptional response to fasting is characterized by widespread changes in enhancer activity displaying altered chromatin accessibility and histone acetylation (3). Thus, we hypothesized that glucagon-corticosterone cooperation will be reflected in altered enhancer dynamics. To explore this, we profiled enhancer dynamics in response to combinatorial hormone treatment (glucagon, corticosterone and a dual treatment) in PMH. Enhancer activity was measured by chromatin immunoprecipitation sequencing (ChIP-seq) of the well-established active histone mark H3K27-acetyl (H3K27ac) (50). Focusing first on regions adjacent to the promoter, we found that regions neighboring glucagon-induced genes show a glucagon-dependent increase in H3K27ac. A similar association between gene induction and H3K27ac levels is observed in corticosterone- and dual-induced genes (Figure 3A). Thus, promoter-proximal regions show increased enhancer activity in response to hormone treatment. This is most prominent in the region closely flanking (∼1 kb) the promoter. Antagonistic genes showed increased H3K27ac in the single treatment while in the dual treatment this increase was significantly dampened. In contrast, the promoter-proximal regions of synergistic genes showed enhancer activation in both single treatments, which was potently augmented in the dual treatment (Figure 3B). The above analysis focused on H3K27ac signal proximal to promoters of hormone-induced genes. To more broadly examine hormone-dependent enhancer activity without distance constraints, we first defined H3K27ac sites genome-wide via ChIP-seq peak-calling (see Methods). Then, we measured a hormone-dependent increase in H3K27ac signal within all H3K27ac sites genome-wide. We found notable enhancer dynamics following hormone treatments. Glucagon led to activation of 1,746 enhancers, corticosterone activated 2,208 enhancers and the dual treatment led to activation of 5,259 enhancers (Supplementary Table S3). Similar to fasting-activated H3K27ac sites (3), most hormone-activated sites (95–97%) were remote from the promoter region, (Supplementary Table S3; promoter proximal sites were defined as sites within -1 kb to + 0.1 kb of the transcription start site, as is commonly accepted). Together, these results show that the cooperation between fasting hormones leads to major alterations in enhancer status which is concordant with the observed gene expression patterns.

To predict which TFs mediate hormone-dependent enhancer activation, we performed de novo motif enrichment analysis on enhancers activated by single and dual treatments. The most enriched motif within glucagon-activated enhancers was the cAMP response element (CRE) bound by CREB. The most enriched motif within corticosterone-activated enhancers was the glucocorticoid response element (GRE) bound by GR. The two most highly enriched motifs in dual-activated enhancers were the GRE and CRE (Supplementary Figure S1). This is in complete agreement with the TFs known to be activated by glucagon and corticosterone – CREB and GR, respectively. These results attest to the functional relevance of the defined enhancer groups and suggest that the cooperation between glucagon and corticosterone in regulating genes is mediated via CREB and GR.

### The glucagon-CREB axis mediates enhancer activation and potentiates GR binding

**Figure 4.**
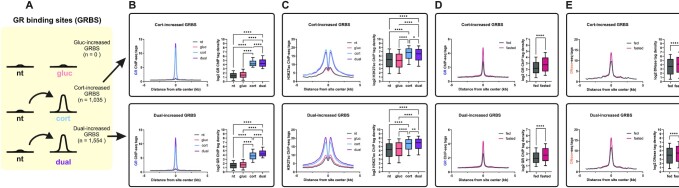
Corticosterone-dependent GR binding is augmented by glucagon. (**A + B**). Quantifying differential binding of GR shows that glucagon treatment alone does not increase the number of GRBS, corticosterone leads to prominent GR activation as measured via increase in GBRS and the dual treatment further augments it (increased GR binding determined as fold change ≥ 1.5, adj. *P* value ≤ 0.05 over the non-treated control). (**C**). Enhancer activity was measured by H3K27ac signal in regions surrounding GRBS. The increase in GR binding is associated with enhancer activation in both the corticosterone and dual treatments. (**D**). GR binding in mouse liver in the fed or fasted conditions was quantified. Corticosterone- and dual-activated GRBS show increased GR binding in mouse liver following fasting [fasting-dependent GR ChIP-seq data was obtained from (3)]. (**E**). Enhancer activity in mouse liver in the fed or fasted conditions was evaluated by chromatin accessibility (as measured by levels of Dnase-seq signal). Corticosterone- and dual-activated GRBS reside within fasting-activated enhancers [fasting-dependent chromatin accessibility data was obtained from (3)]. (nt – non-treated; gluc – glucagon; cort – corticosterone).

Due to the prevalence of both TF motifs within hormone-activated enhancer regions, we hypothesized that the synergy between glucagon and corticosterone is mediated by a synergistic cooperation between CREB and GR within enhancers. We postulated that this cooperation is brought about via assisted loading within enhancers (see Introduction) as this cooperative model was previously shown to promote synergistic gene expression (31,32). In a previous report we showed that GR assists CREB binding next to gluconeogenic genes (3). However, the enhancer environment, the motif determinants and the genome-wide transcriptional outcomes of GR-CREB assisted loading were never explored. Importantly, while CREB binding was shown to be assisted by GR at some sites, the binding pattern of GR and whether it changes between the single and dual treatments is unknown. Due to the enhancer activation patterns and motif enrichment analyses (Figure 3, Supplementary Table S3), we hypothesized that CREB-GR assisted loading is bi-lateral. I.e., at some enhancers GR assists CREB loading while in others, CREB assists GR loading. This is an unexplored notion of TF cooperativity and we tackled it by performing ChIP-seq for GR in all four conditions. As expected, following both corticosterone and the dual treatment, more GR binding sites (GRBS) were detected (Supplementary Table. S4) and GR binding intensity was markedly increased (Figure 4A). Moreover, de novo motif enrichment analysis revealed the GRE as the top enriched motif in GRBS found in the corticosterone and the dual treatments, while the GRE was missing in the non-treated or glucagon-treated conditions (Supplementary Table. S4). These results show that, as expected, noteworthy GR binding to the genome occurs only following corticosterone stimulation and is mediated by binding to GREs.

To examine a possible promoting effect of glucagon on GR binding, we evaluated dynamic GR binding by determining differential binding following hormone treatment using fold change and p value cutoffs as compared to the non-treated control. There were no sites showing increased GR binding following glucagon treatment. This suggests that, as expected (and as shown further below), glucagon does not directly stimulate GR binding. In contrast, 1,035 sites showed increased GR binding following corticosterone treatment, aligning well with known GR biology and with the potent effect corticosterone had on gene expression in PMH (Figure 1). Remarkably, 1,554 GRBS showed an increase following the dual treatment, 636 of which were not increased in the single corticosterone treatment (Figure 4A, B, Supplementary Table S5). The marked increase in GR binding is associated with a concordant increase in enhancer activity around the GRBS (Figure 4C). Importantly, the sites where GR binding was increased in corticosterone- or dual-treated cells also show increased GR binding and enhancer activity in mouse liver following fasting, suggesting these sites are functionally relevant not only in corticosterone-treated PMH but also during fasting in vivo [Figure 4D, E; fasting-dependent chromatin accessibility and GR ChIP-seq data was obtained from (3)]

**Figure 5.**
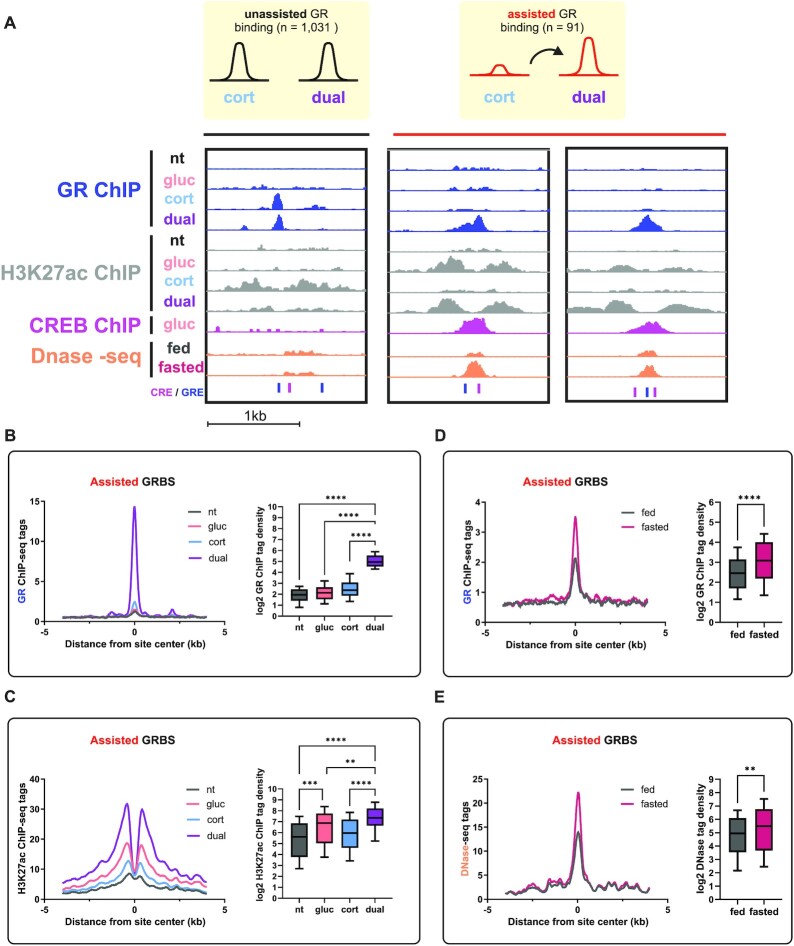
GR binding is prominently increased by glucagon in an enhancer-specific manner. (**A + B**). Quantifying differential binding of GR shows that although glucagon alone does not affect GR binding (Figure 4A, B), glucagon significantly increases corticosterone-dependent GR binding in certain GRBS termed ‘assisted sites’. Example GRBS are shown in genome browser tracks together with H3K27ac, CREB binding, chromatin accessibility as well as CRE and GRE occurrence. (**C**). Enhancer activity was measured by H3K27ac signal in regions surrounding GRBS. Glucagon treatment alone led to significant enhancer activation around assisted GRBS, while it did not affect unassisted GRBS (compare with Figure 4C). (**D**). GR binding in mouse liver in the fed or fasted conditions was quantified. Assisted GRBS show increased GR binding in mouse liver following fasting [fasting-dependent GR ChIP-seq data was obtained from (3)]. (**E**). Enhancer activity was evaluated by chromatin accessibility (as measured by increased Dnase-seq signal) in regions surrounding GRBS. Assisted GRBS reside within fasting-activated enhancers [fasting-dependent chromatin accessibility data was obtained from (3)].

The observation that 636 GRBS are found only in the dual treatment suggests that GR binding is substantially increased in the presence of glucagon, presumably due to assisted loading. To show this effect from a different angle and to substantiate it statistically, we performed a differential GR binding comparison directly between the corticosterone and the dual treatments. We found 91 GRBS in which GR binding was significantly increased in the dual treatment compared to corticosterone alone (Figure 5A, Supplementary Table S5). Therefore, we have shown in two separate analyses that corticosterone-dependent GR binding is significantly increased in the presence of glucagon. The difference in the number of glucagon-augmented GRBS between the two different analyses (636 vs. 91) is due to the stringency of the direct comparison between corticosterone-treated and dual-treated cells. Due to the higher reliability of the direct comparison, we chose to focus on this group of sites where glucagon strongly assists GR binding. Thus, the 91 sites where GR binding was significantly higher in the dual treatment compared to corticosterone treatment were termed ‘assisted sites’. In contrast, sites where GR binding is unaffected by glucagon (i.e. it is indistinguishable between corticosterone and the dual treatments) were termed ‘unassisted sites’ (Figure 5A). We found that unassisted sited comprise the vast majority of corticosterone-increased sites (99.6%; 1,031/1,035, Supplementary Table S5). Therefore, all further analyses of assisted sites are compared to corticosterone-increased GRBS (Figure 4).

Interestingly, assisted sites do not reach GR binding intensities that are higher than unassisted sites. Rather, only in the presence of glucagon do assisted sites reach comparable levels of unassisted sited (Figure 5B, compare to Figure 4B). Thus, in unassisted sites GR is able to optimally bind without help while in assisted sites, maximal GR binding is only achieved with the help of glucagon. Assisted loading is a TF crosstalk model in which direct protein-protein interaction between the two TFs is not required. Instead, the model is based on one TF activating the enhancer, thereby allowing better access to it by the second TF. In accordance with the model, we found that H3K27ac is increased in assisted sites following glucagon treatment alone while in unassisted sites glucagon has no effect on enhancer activity (Figure 5C, compare to Figure 4C). This is consistent with a scenario in which a glucagon-activated TF, leads to enhancer activation, permitting subsequent GR binding. In line with the PMH data, GR binding and enhancer activity in assisted sites is increased during fasting in mouse liver [Figure 5D, E; fasting-dependent GR ChIP-seq and chromatin accessibility data was obtained from (3)], suggesting the activation of these enhancers is pertinent during fasting in vivo. Of note, enhancers harboring assisted sites are more accessible than enhancers harboring unassisted sites (Figure 5E, compare to Figure 4E). Finally, we found that the nearest gene to 43% of assisted sites is a dual-induced gene, half of which are also synergistically-induced. This is in contrast to only 8% of assisted sites whose nearest gene is a corticosterone-induced gene (Supplementary Table S5). Considering that a ‘nearest gene’ analysis often underestimates the true number of regulated genes, we conclude that assisted sites are associated with the combined transcriptional program of glucagon and corticosterone. Taken together, these findings show that glucagon alone activates enhancers harboring assisted GRBS and potentiates GR binding there. Also, while GR can optimally bind unassisted sites following corticosterone, only following a dual treatment does GR optimally bind assisted sites at a level comparable to unassisted sites.

**Figure 6.**
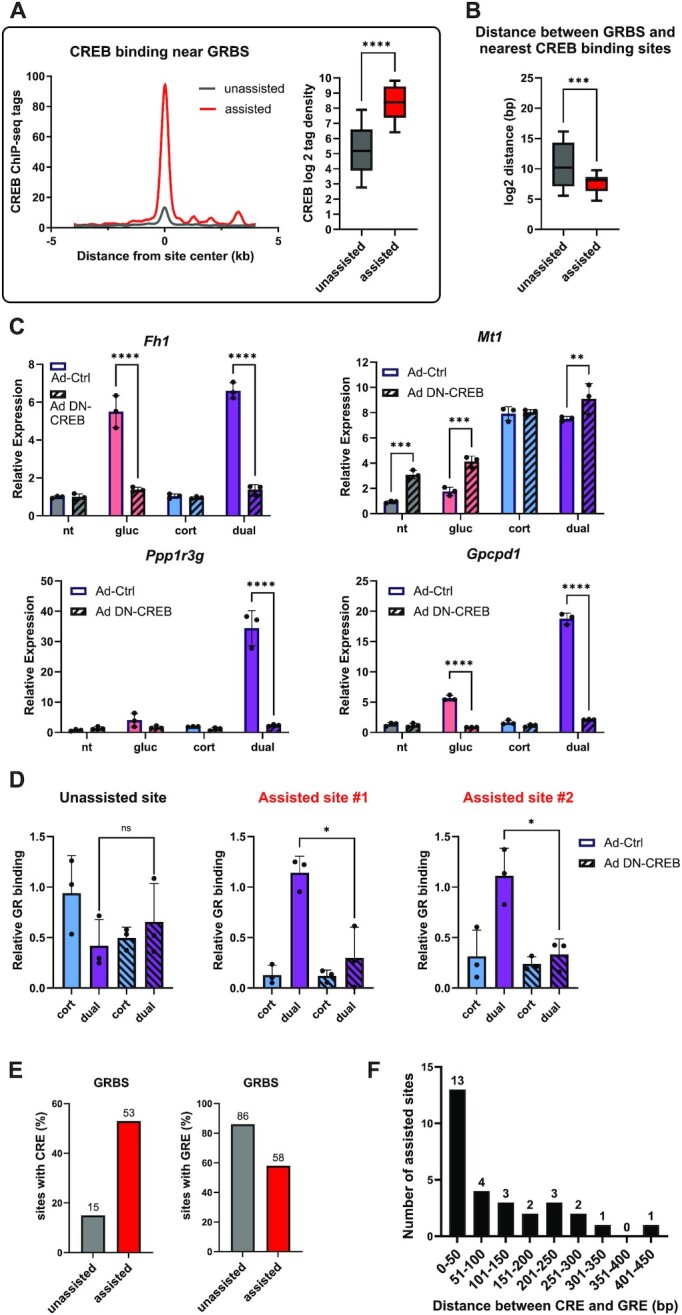
CREB assists the loading of GR following glucagon treatment. (**A**). Quantifying differential binding of CREB near GRBS shows preferential CREB binding near assisted sites. (**B**). Measuring the distance between GRBS and CREB binding sites shows that CREB tends to bind closer to assisted sites. (**C**). Primary mouse hepatocytes were infected with adenovirus expressing either DN-CREB (Ad-DN-CREB) or a control (Ad-Ctrl). After 24 h, cells were treated with the indicated hormones for 3 h. Gene expression was measured by qPCR. Ad-DN-CREB abolished glucagon-dependent induction which is unaffected by corticosterone (*Fh1*) as well as synergistic gene induction (*Ppp1r3g* and *Gpcpd1*). (**D**). Primary mouse hepatocytes were infected with adenovirus expressing either DN-CREB (Ad-DN-CREB) or a control (Ad-Ctrl). After 24 h, cells were treated with the indicated hormones for 1 h followed by GR ChIP. GR binding was measured via qPCR. (**E**). Scanning motif occurrences in assisted and unassisted GRBS shows that the percentage of the CRE increases in assisted sites while GRE occurrences decrease in assisted sites as compared to unassisted sites. (**F**). Measuring the distance between CREs and GREs in enhancers harboring assisted GRBS shows no fixed inter-motif distance. (nt – non-treated; gluc – glucagon; cort – corticosterone).

To eliminate secondary effects, all ChIP-seq experiments in this study where done after only 1 h of treatment. Thus, it is unlikely that the stimulatory effect of glucagon on GR depends on secondary effects of glucagon target genes. Rather, a crosstalk between glucagon and corticosterone downstream signaling is the probable cause. The effect of glucagon on GR binding is enhancer-specific (i.e. occurs only in assisted sites and not on unassisted sites). Nonetheless, to exclude a global effect of glucagon signaling on GR activity, we examined GR mRNA and protein levels as well as GR nuclear localization. We found that the mRNA or protein levels of GR (or CREB) where not increased in the dual treatment compared to single treatments (Supplementary Figure S2A, B). Also, glucagon did not augment corticosterone-dependent GR nuclear localization (Supplementary Figure S2C). Therefore, the effect of glucagon on GR is restricted to assisted sites rather than generally affecting GR activity. In support of this, de novo motif enrichment analysis revealed that in unassisted sites, GRE was the top enriched motif while CRE was absent. In stark contrast, the two most enriched motifs within assisted sites were CRE and GRE (Supplementary Figure S3), associating CREB in assisted loading of GR. In accordance, the binding of CREB following glucagon treatment was very prominent near assisted sites while it was nearly undetected in unassisted sites [Figures 5A, 6A; CREB ChIP-seq data was obtained from (3)]. In addition, CREB binding sites were significantly closer to assisted GRBS as compared to unassisted sites (Figure 6B). This further implicates CREB in glucagon-mediated assisted loading. To show that the glucagon-CREB axis is responsible for synergistic gene expression, we infected cells with an adenovirus expressing a dominant negative peptide against CREB [DN-CREB, (51)]. As expected, DN-CREB negated glucagon-dependent induction of a CREB target gene (*Fh1*) while it did not affect the corticosterone-dependent induction of a known GR target gene (*Mt1*). In contrast, we found that DN-CREB abolished synergistic gene induction altogether (*Ppp1r3g* and *Gpcpd1*; Figure 6C). Importantly, we measured GR binding by ChIP and found that binding of GR at an unassisted site was unaltered by DN-CREB while GR occupancy at assisted sites was abolished by DN-CREB (Figure 6D).

To find the percentage of GRBSs that harbor GRE or CRE motifs, we searched for motif occurrence in assisted vs. unassisted sites. Indeed, the occurrence of the CRE more than tripled in assisted sited compared to unassisted sites. Remarkably, the occurrence of the GRE was reduced in assisted sites (Figure 6E). We hypothesized that this reduction is due to assisted sites harboring GREs that are less similar to the consensus GRE, leading to them not being identified in the targeted motif search. In other words, we reasoned that the difference in GRE occurrence found between assisted and unassisted sites reflects a feature of assisted sites – these sites contain a ‘weaker’ GRE. To explore this, we examined the GRE motif score given in the de novo motif enrichment analysis. The closer the motif score is to 1, the closer it is to the consensus motif. We found that the GRE motif score in unassisted sites is 0.89 while in assisted sites the score is 0.78 (Supplementary Figure S3). Thus, unassisted sites harbor a ‘stronger’ motif with higher affinity to GR while assisted sites harbor a GRE more distant from the consensus with reduced affinity to GR. This finding supports the concept that in assisted sites, GR binding to weaker motifs is facilitated by CREB which activates the enhancers, making them more amenable to binding, thereby improving the low incidence of GR binding events to a weaker motif. This interpretation is in line with the observation that assisted sites show weaker GR binding in the corticosterone treatment but in the dual treatment reach binding intensity comparable to unassisted sites (Compare Figures 4B to 5B).

The assisted loading model assumes one TF (in this case CREB) facilitates the binding of another TF (in this case GR) by enhancer activation and irrespective of direct TF-TF interaction. According to this assumption and in contrast to TF-TF heterodimerization, the motifs for both TFs could be placed tens or even hundreds of nucleotides apart. To explore this, we isolated assisted sited where both a GRE and a CRE were found and binned them by the distance between the two motifs. We found no preference to a fixed number of nucleotides spacing the two motifs [as is the case in heterodimerization (52)]. Rather, motif distance spanned between 10 and 421 nucleotides, with a roughly even distribution at 100 nucleotides and upwards (Figure 6F). Taken together, these findings reveal that glucagon leads to enhancer activation by CREB binding, which assists GR binding. In the lack of a glucagon signal and enhancer activation, GR binding is abolished or significantly diminished due to inaccessibility to the enhancer harboring a weaker GRE.

### Assisted loading is operative within enhancer clusters

Various studies in recent years have shown that enhancers often appear in clusters whereby several enhancers (which we term in the rest of the text ‘enhancer units’) flank each other and are concordant in histone modification and chromatin accessibility patterns (53). Enhancer units within a cluster often cooperate in regulating a single gene (53). The enhancer cluster phenomenon is convincingly explained by the finding that enhancer clusters are physically proximal in the three-dimensional space, thereby assembling one regulatory apparatus that promotes gene transcription. The aggregated enhancer units are all positioned within the same chromatin ‘loop’ which is bordered by CTCF (54) and together create an environment more permissive toward transcription by recruitment of co-regulators and chromatin factors (55). Thus, the concept of enhancer activation can be expanded to enhancer *cluster* activation. By proxy, assisted loading can occur within an enhancer cluster whereby one TF binds one enhancer unit within the cluster, leading to activation of the enhancer cluster at large. Then, the other TF more easily binds a second enhancer unit within the same cluster. This hypothesis might reconcile an unexpected observation – only 32% of assisted sites harbored both a CRE and a GRE.

**Figure 7.**
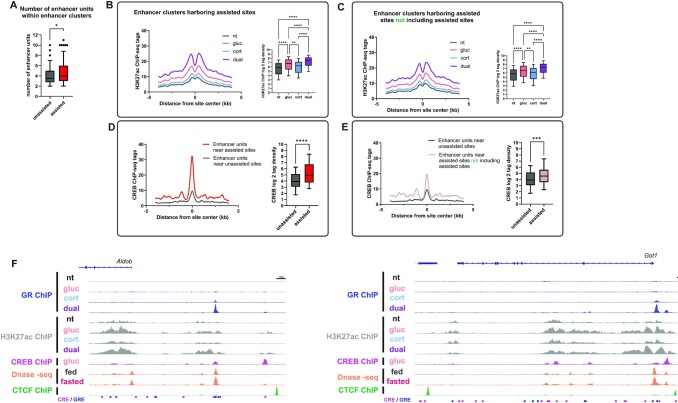
Assisted loading of GR occurs within enhancer clusters, driving synergistic gene expression. (**A**). Quantification of enhancer units within enhancer clusters shows that assisted sites reside within enhancer clusters containing a higher number of enhancer units compared to unassisted sites. (**B + C**). Enhancer activity was measured by H3K27ac signal in enhancer units within enhancer clusters. Glucagon treatment alone led to significant enhancer activation within enhancer units around assisted GRBS. The effect of glucagon was evident even after excluding the enhancer unit harboring assisted GRBS, showing that the effect of glucagon spans across the cluster and not only in the specific enhancer unit. (**D + E**). CREB binding was measured in enhancer units within enhancer clusters. Glucagon treatment alone led to significant CREB binding within enhancer units around assisted GRBS. The effect of glucagon is evident even after excluding the enhancer unit harboring assisted GRBS, showing that the effect of glucagon spans across the cluster and not only in the specific enhancer unit. (**F**). Genome browser tracks of synergistically-induced genes shows enhancer clusters broadly activated by glucagon. These clusters harbor CREs, GREs, CREB binding, assisted GR binding as well as fasting-activated enhancers. Enhancer clusters are flanked by CTCF. (nt – non-treated; gluc – glucagon; cort – corticosterone).

To examine this hypothesis, we scanned assisted sites for nearby enhancers, using chromatin accessibility data as an acceptable marker for enhancers (56). Based on previous conventions (57), we scanned a 12.5kb region upstream and downstream of assisted sites for enhancer clusters. We excluded assisted sites in the vicinity of which no enhancers were detected (n = 13). Most of the remaining sites resided in clusters containing multiple enhancers, up to 11 enhancers within a cluster (Figure 7A). We then tested for the co-occurrence of GRE and CRE within enhancer clusters, revealing that 61% of clusters contain both a GRE and a CRE. Thus, while motif co-occurrence within a single enhancer harboring an assisted site occurs in 32% of assisted sites, almost two thirds of enhancer clusters harbor both a CRE and a GRE. Unassisted sites also tended to reside within clusters but in contrast to assisted sites, these clusters contained less enhancer units (Figure 7A). Enhancer clusters harboring unassisted sites contained less co-occurrence events where both a CRE and a GRE were detected (50%). To check if glucagon is able to increase the activity of enhancer clusters at large (and not only individual enhancer units harboring assisted sites), we evaluated enhancer activity of all enhancer units within the clusters. A single treatment of glucagon led to robust activation of enhancer units within enhancer clusters (Figure 7B). Notably, this activation was evident also after exclusion of enhancer units harboring assisted sites (Figure 7C). In accordance, we found that CREB binding following glucagon treatment is increased in enhancer units across clusters harboring assisted sites when compared to clusters harboring unassisted sites (Figure 7D). Importantly, CREB binding was also increased even when excluding enhancer units harboring assisted sites (Figure 7E). Thus, glucagon-dependent enhancer activation and CREB binding are observed across enhancer units of clusters where assisted loading is found and not only in the individual enhancer unit harboring the assisted site. Enhancer clusters often reside near synergistically-induced genes. Examples are shown in Figure 7F and Supplementary Figure S4 where the loci of five synergistic genes are depicted, showing glucagon-dependent CREB binding in the cluster, glucagon-dependent enhancer activation across the cluster and assisted GR binding. This occurs in sites with co-occurrence of CREs and GREs and is associated with fasting-dependent increase in enhancer activity. Moreover, only the edges of enhancer clusters are flanked by CTCF while there is no CTCF binding within a cluster. This further suggests that the units within enhancer clusters function together to regulate gene expression and are proximally-located in three-dimensional space, positioned within the same chromatin loop (Figure 7F, Supplementary Figure S4). In summary, glucagon leads to CREB binding and enhancer activation across the cluster. In the dual treatment this potentiates GR binding, leading to synergistic gene induction.

## DISCUSSION

Cells are routinely exposed to a myriad of extracellular signals and are constantly responding to them, thereby adapting to a changing environment and maintaining homeostasis. The manner by which extracellular signals are integrated and translated in cells to produce a coherent response is poorly understood. Here, we investigated the hepatic fasting response which is heavily reliant on transcriptional regulation (14,23,58). This response is controlled by the combinatorial effect of several signals, chief among them are glucagon and glucocorticoids. We treated PMH with different combinations of hormones and analyzed the transcriptional response to these treatments as well as the dynamics in enhancer activation imposed by them. We found that the crosstalk between glucagon and glucocorticoids is not monotonic. Rather, the two hormones cooperate to synergistically induce some genes while antagonize each other's response in different sets of genes. Synergy and antagonism are accompanied with corresponding enhancer activity in the gene loci. Thus, the crosstalk between the two hormones is gene-specific and enhancer-specific and is not uniform across the genome. The complex crosstalk between the two hormones could be a mechanism for a tailored response: glucocorticoids increase not only during fasting but following various kinds of stress (59). Thus, combinations of glucocorticoids with other signals could together produce a response more specific to the particular stress. Indeed, we found that antagonized genes have roles unrelated to the fasting response. In contrast, synergistically-induced genes play key roles in the fasting response in various functions supporting gluconeogenesis. These findings suggest that in contrast to single treatments, integration of extracellular signals leads to a tailored response addressing the specific gene programs needed to maintain homeostasis.

Synergy between GR and the cAMP-PKA pathway in regulating gene expression was shown (3,19,26–29,60,61). However, the mechanism behind it is unclear. Several reports show that the cAMP-PKA pathway augment GR in diverse manners: cAMP was shown to induce transcription of the GR gene (62,63) and increase its mRNA stability (64,65). PKA was shown to phosphorylate GR (66,67) and a physical interaction between GR and CREB was reported (26,62) the glucagon-cAMP-PKA pathway was shown to induce the gene levels of two GR co-activators – PGC1α (27,68) and CRTC2 (29). Taken on face value, these types of crosstalk seem to provide a possible explanation to the augmenting effect of glucagon on GR-dependent gene expression which we have observed. However, we assert that these effects cannot reconcile our observation for several reasons: **a)** We observe no glucagon-dependent GR gene induction, increase in GR protein levels or increase in GR nuclear localization. **b)** The effect of glucagon on GR was highly enhancer-specific. Most GRBS were unaffected by glucagon altogether. This shows that the augmenting effect of glucagon is brought about only at particular enhancers and an effect of glucagon on overall GR activity as previously described could not explain this observation. **c)** Inhibiting CREB does not affect GR target genes but do abolish synergistic gene induction. **d)** CREB inhibition does not affect GR binding at unassisted sites but does impair it at assisted sites. **e)** We observed both a synergistic and an antagonistic crosstalk between glucagon and corticosterone simultaneously. This bifurcated response could not be explained by an overall effect of glucagon on GR. **f)** We did not find a fixed motif distance between CREs and GREs, making the physical interaction scenario between GR and CREB less likely in our case.

We found that the glucagon-CREB axis leads to enhancer activation that assists GR binding near synergistically-induced genes. We have previously shown the reciprocal phenomenon whereby GR assists the loading of CREB in a different set of enhancers (3). Thus, the synergistic crosstalk between glucagon and glucocorticoids is driven by ‘bi-lateral assisted loading’ in which certain enhancers are activated by CREB, assisting the loading of GR while other enhancers are activated by GR, assisting the loading of CREB. We provide evidence that the assisting TF (i. e. the TF binding the enhancer first and activating it) is the factor whose motif has higher affinity its corresponding TF. The assisted TF is the factor with a weaker motif, therefore it can only reach optimal binding following enhancer activation and increased accessibility. Therefore, in a bi-lateral assisted loading model, two TFs maximize a biological response by coordinating optimal binding that is only achieved in the presence of the two signals.

The assisted loading mode of cooperation between TFs is particularly fitted for tailoring a transcriptional response to two or more signals because it is gene-specific and enhancer-specific. Thus, while other modes of cooperation affect all genes regulated by a certain TF, assisted loading leads to synergistic gene induction only on a subset of genes. It appears that GR serve as an extreme example for assisted loading-type of crosstalk with other TFs. GR binding to the genome was shown to be altered by several TFs under different conditions. GR occupancy is increased by C/EBPβ (69), STAT5 (70), E47 (71), AP-1 (72) and CREB as shown here. Reciprocally, GR was found to assist the binding of TFs such as FoxA (73,74) and CREB (3). Presumably, this high degree of cooperation with various TFs stems from the fact that GR is expressed in virtually all cell types and is activated by glucocorticoids which are secreted following a myriad of stress situations. Therefore, in order to respond to different types of stress in different cell types and under different circumstances, the glucocorticoid stress signal is integrated with more specific signals via GR assisted loading. Fitting with this scenario, GR binding was found to be increased by STAT5 in livers of mice under a high fat diet, presumably due to increased growth hormone signaling in these mice (70). Thus, it is plausible to assume that GR assisted loading is not restricted to gluconeogenesis but also plays a role in other biological programs.

Our data suggest that assisted loading is not restricted to two TFs binding at the same enhancer. Rather, assisted loading occurs across enhancer units within enhancer clusters. Clusters of enhancers have emerged as a central mode of gene regulation in which several enhancer units share enhancer characteristics (chromatin accessibility, histone modifications) and together regulate gene expression. Enhancer units within the cluster are in close proximity in three-dimensional space and thus constitute a regulatory apparatus promoting transcription. Recent observations suggest that TF binding is controlled by activation of enhancer clusters rather than single enhancers (75). Moreover, it was recently shown that the binding of a TF at one enhancer unit leads to increased binding of the same TF at other units (76). Here, we show that one TF augments the binding of a *different* TF activated by a different hormone. Thus, two different hormonal signals are integrated by two different TFs via assisted loading: glucagon activates CREB and increases enhancer activity of the cluster at large, thereby assisting binding of GR stimulated by corticosterone. Based on these results, it is plausible that glucagon, via CREB, generates an environment conducive to GR binding by increased enhancer cluster acetylation. Cluster-wide histone acetylation was shown to positively affect gene induction via several possible mechanisms (55). Our results point to a scenario whereby the glucagon-CREB axis activates enhancer clusters, assists GR loading to them, eventually leading to synergistic gene expression.

In summary, in this study we provide evidence that by employing assisted loading, two signals are integrated to boost a biological response by coordinating enhancer activation, TF binding and synergistic gene expression. This phenomenon is bi-lateral and is dictated by motif strength, serving to optimize gene regulation by enhancers. This integration of signals expands beyond single enhancers and is also at play in enhancer clusters.

## DATA AVAILABILITY

All RNA-seq and ChIP-seq data have been deposited in the Gene Expression Omnibus (GEO; https://www.ncbi.nlm.nih.gov/geo/) under accession number GSE189271.

## Supplementary Material

gkac358_Supplemental_Files
