# Supplementary material for: Hormone-controlled cooperative binding of transcription factors drives synergistic induction of fasting-regulated genes
Source: Nucleic Acids Res. 2022 May 12;50(10):5528–44. doi: 10.1093/nar/gkac358 (PMC9177981; doi:10.1093/nar/gkac358)
Supplement: gkac358_Supplemental_Files [file gkac358_supplemental_files.zip › Supplementary Figures.pdf]

<u>***Supplementary Figures***</u>

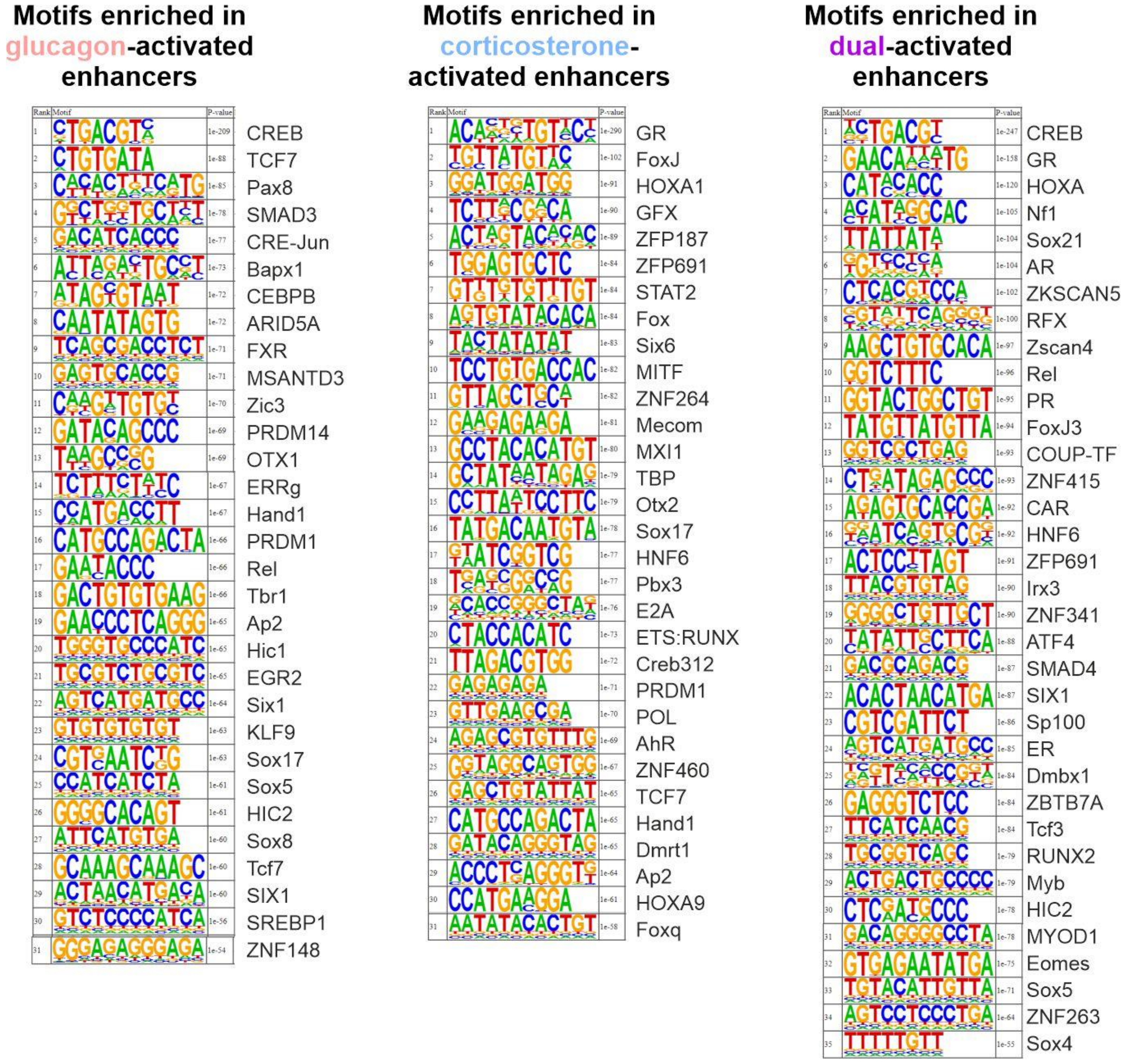

Supplementary Figure 1: Glucagon-activated enhancers are enriched with CREs and corticosterone-activated enhancers are enriched with GREs

De novo motif enrichment analysis reveals enriched motifs in hormone-activated enhancers. The TF indicated next to the motif is the TF whose motif most resembles the de novo-found motif. All motifs with p value ≤ 1^{-10} are shown.

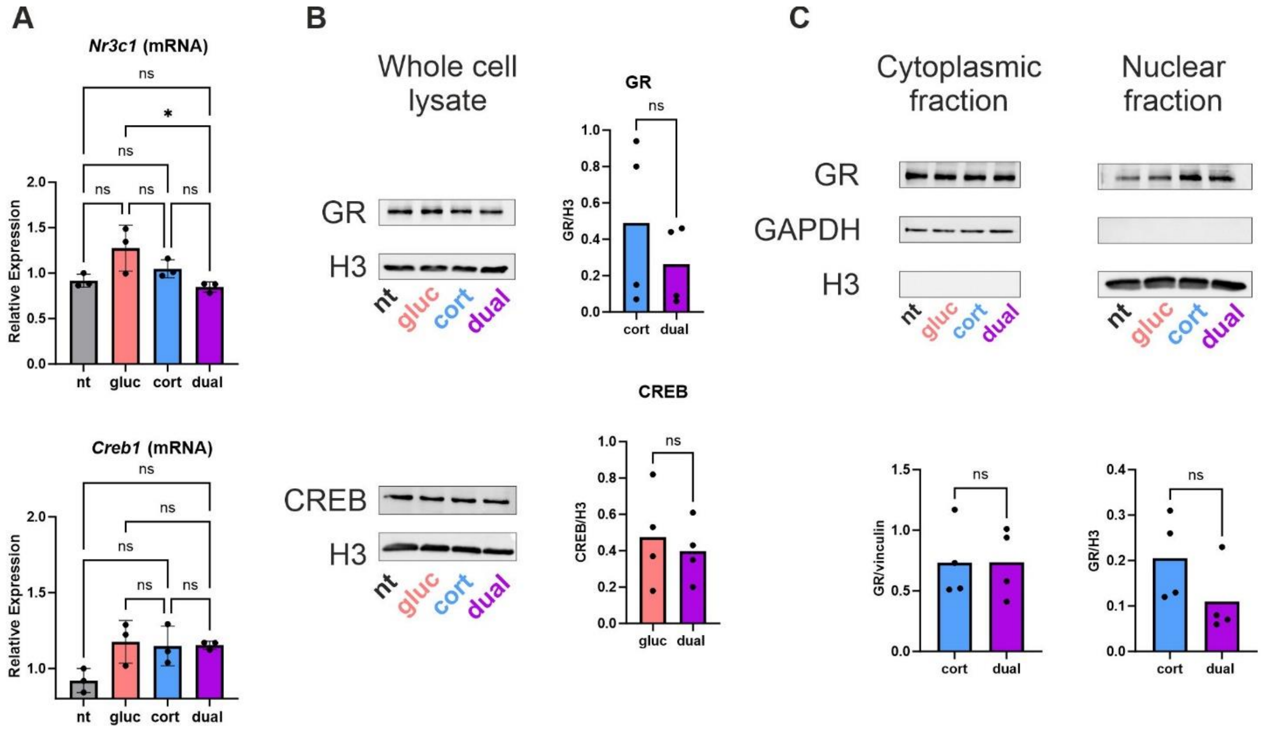

Supplementary Figure 2: GR and CREB levels are unaffected by hormone treatments

- **A.** PMH were treated with indicated hormones for 3 h. The mRNA levels of GR (*Nr3c1*) and CREB (*Creb1*) were measured by quantitative PCR.
- **B.** PMH were treated with indicated hormones for 3 h. The protein levels of GR and CREB were measured by quantitative western blot.
- **C.** PMH were treated with indicated hormones for 3 h. The nuclear and cytoplasmic levels of GR were measured by cellular fractionation followed by western blot.

(nt – non-treated; gluc – glucagon; cort – corticosterone; GAPDH - glyceraldehyde 3-phosphate dehydrogenase)

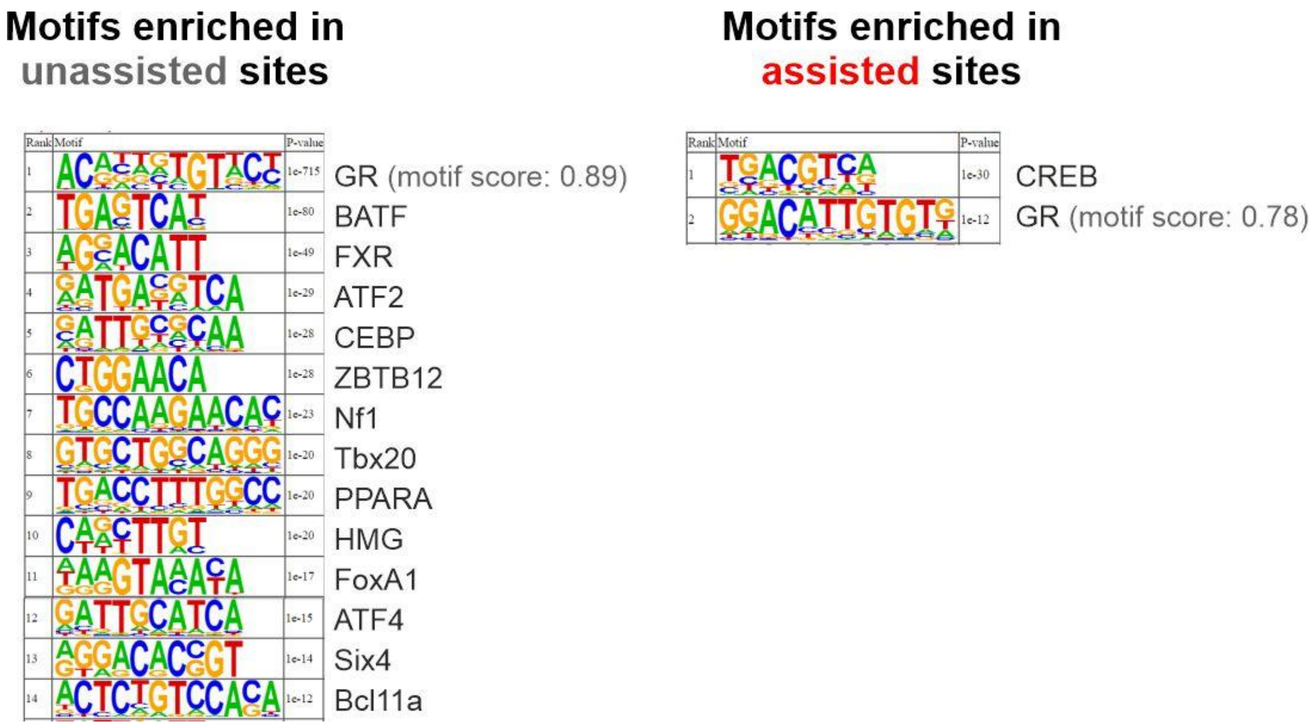

Supplementary Figure 3: The CRE is enriched in assisted GRBS, in contrast to unassisted GRBS
De novo motif enrichment analysis reveals enriched motifs in assisted and unassisted GRBS. The TF indicated next to the motif is the TF whose motif most resembles the de novo-found motif. The motif score of the GRE represents its similarity to the consensus GRE (higher score = higher similarity). All motifs with p value ≤ 1^{-10} are shown.

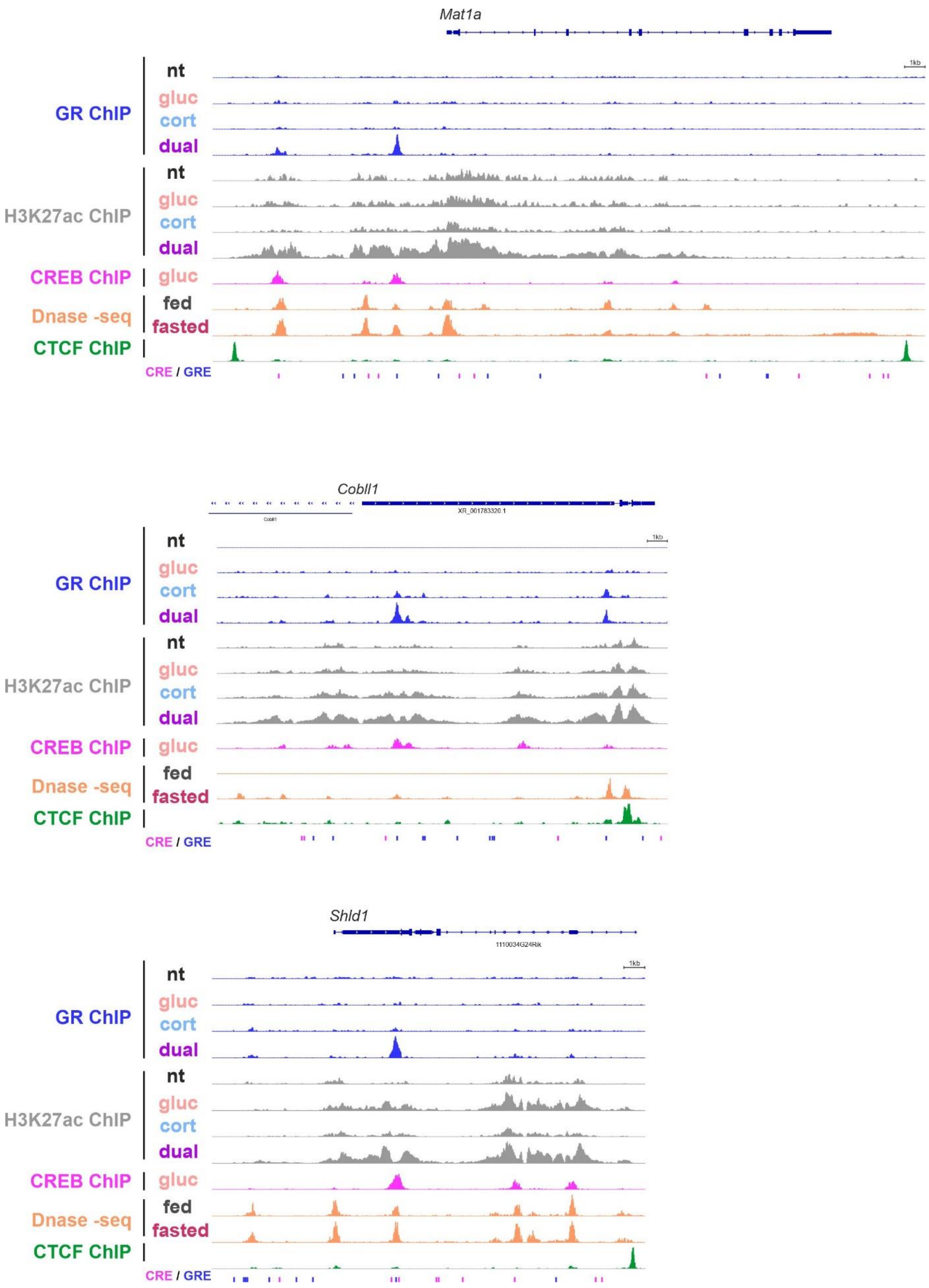

Supplementary Figure 4: Genome browser tracks of synergistically-induced genes' loci show enhancer cluster activation. These clusters harbor CREs, GREs, CREB binding, assisted GR binding as well as fasting-activated enhancers. Enhancer clusters are flanked by CTCF.

Supplementary Table captions

Supplementary Table 1: Differentially expressed genes full lists

Supplementary Table 2: Pathway enrichment analyses of synergistic and antagonistic genes

Supplementary Table 3: Hormone-activated H3K27ac sites

Supplementary Table 4: GR binding sites and enriched motifs

Supplementary Table 5: Hormone-activated GR binding sites
